# Phytochemical Study, Cytotoxicity, and Genotoxicity of the Methanolic Extract of *Geranium diffusum* Kunth

**DOI:** 10.3390/plants14050777

**Published:** 2025-03-03

**Authors:** Juan Carlos Romero-Benavides, Tatiana Añazco-Loayza, Anabel Correa-Sinche, Andrea Alvarez-Ruiz, Luis Miguel Guamán-Ortiz, Rodrigo Duarte-Casar, Natalia Bailon-Moscoso

**Affiliations:** 1 Departamento de Química, Facultad de Ciencias Exactas y Naturales, Universidad Técnica Particular de Loja, Loja 110108, Ecuador; tlanazco@utpl.edu.ec; 2 Carrera de Bioquímica y Farmacia, Facultad de Ciencias Exactas y Naturales, Universidad Técnica Particular de Loja, Loja 110108, Ecuador; accorrea2@utpl.edu.ec (A.C.-S.); acalvarez4@utpl.edu.ec (A.A.-R.); 3 Facultad de Ciencias de la Salud, Universidad Técnica Particular de Loja, Loja 110108, Ecuador; lmguaman@utpl.edu.ec (L.M.G.-O.); ncbailon@utpl.edu.ec (N.B.-M.); 4 Tecnología Superior en Gestión Culinaria, Pontificia Universidad Católica del Ecuador—Sede Manabí, Portoviejo 130103, Ecuador; rduarte@pucesm.edu.ec

**Keywords:** flavonoids, Ecuadorian medicinal plants, cytotoxicity

## Abstract

*Geranium diffusum* Kunth is a medicinal plant native to Ecuadorian highlands with limited scientific study despite its ethnopharmacological relevance. The present study investigates the phytochemical properties and cytotoxic effects of the methanolic extract of *G. diffusum*. Comprehensive analyses revealed a rich composition of bioactive compounds, predominantly flavonoids and rutin, isoquercetin, and isorhamnetin-3-glucoside, known for their therapeutic potential, were isolated. The extract and its solvent fractions were evaluated for cytotoxic activity against three cell lines: RKO, SW613-B3, and HCT-116. Notably, the ethyl acetate fraction exhibited cytotoxicity with an IC_50_ value of 44.47 ± 1.02 μg/mL against the SW613-B3 cell line, indicating its significant anticancer activity. The dichloromethane fraction also demonstrated noteworthy effects on the RKO and HCT-116 lines, while the methanolic fraction exhibited higher viability in HCT-116 cells. No genotoxicity was found in CHO-K1 cells. These findings underscore the potential of *Geranium diffusum* as a valuable source of bioactive compounds for developing therapeutic agents against cancer and highlight the need for further research into its pharmacokinetics, in vivo efficacy, and the synergistic effects of its flavonoid constituents. This study contributes to enhancing our understanding of underexplored medicinal plants and supports conservation efforts for Ecuadorian biodiversity.

## 1. Introduction

The use of medicinal plants is as old as humanity, if not older as it may have arisen through imitation of zoopharmacognosy and may be considered at the root of medicine [1]. Around ten percent of vascular plant species are used as medicinal plants, which puts the medicinal plant species in the tens of thousands [2]. The refinement of the trial-and-error use of plants and other therapeutic techniques became traditional medicine, defined by the World Health Organization (WHO) as the sum of skills and practices used in the maintenance of health [3].

Many medicinal plants that have traditionally been used have still not been systematically and scientifically studied. This is particularly evident in South America, where the most known medicinal species are the biologically active coca (*Erythroxylon coca*) and Ayahuasca (*Banisteriopsis* spp. plus *Psychotria viridis*), with many lesser-known species awaiting detailed study [4]. The already studied species frequently require more comprehensive studies as to their activity, phytochemical composition, and ways to develop new treatments or drugs from them [5,6].

Ecuador is the most biodiverse country in the world by surface, a megadiverse South American country that contains several climates and landscapes, from coastal semidesert to the highland plateau of the Andes, to the Amazon basin and the Galapagos islands; it is the “country of the four worlds”, and thus has a biodiversity that is constantly being rediscovered for food and medicine [7,8]. Specifically the plant biodiversity is very high, and its species have been known and used for a long time by Indigenous inhabitants [9].

The traditional use of medicinal plants in Ecuador is an integral part of the culture of its many people, who harvest, cultivate, and sell medicinal plants as part of the indigenous health system [10]. The variety of medicinal plants is so wide that their study is ongoing, and at the time there is a variety of less studied plants. This situation has been addressed in the literature and studies are constantly reducing this research gap [7,11]. Plant species that serve as food or are part of traditional beverages seem to have garnered more attention than non-food medicinal plants [12,13,14,15]. A focus in non-food medicinal species will reduce the gap of poorly studied medicinal plants in Ecuador.

The Saraguro are a close-knit Kichua people that is considered to have been forced to migrate from the Bolivian highlands to Ecuador under the Inca empire [16,17]. Their territory is at the northern end of what has been called “a ‘health axis’ of Andean ethnomedicine” that extends from the Loja province in Ecuador to the department of Piura in northern Peru, where a great biodiversity joins a shared indigenous Andean health system which may have been influenced by the Greek humoral theory of disease through the Spaniard invaders [18]. Saraguros possess intimate knowledge of the medicinal properties and uses of the rich plant diversity of their land, many of which have not yet been studied with current methods, and hold a wealth of knowledge and potential treatments and drugs [16,19,20].

Among these species, one that is present in the southern Ecuador Saraguro ethnobotanical lore is *Geranium diffusum* in the *Geranium* genus. The Saraguro ethnomedicinal system employs a variety of medicinal plants by healers and midwives: the *Geranium* genus is represented in these practices and *G. diffusum* is used by midwives.

The genus *Geranium* Tourn. ex L. comprises 350 species distributed globally, absent only in the poles, arid deserts, and low altitude tropical areas [21]. South America is the region where species of the genus are most abundant, with 100 species [22]. The 46 species in this genus are widely used in horticulture, pharmacy, and research [23].

The *Geranium* species that grow in Ecuador appear mostly as perennial herbs and small woody shrubs, which grow in the Andes mountains between 2500 and 4500 m altitude. Taxonomically, these plants are little known; in Ecuador it is considered that there are 23 species of this genus, of which eight are endemic, two are endangered, and six are vulnerable [24]. Several species of the genus are part of the Ecuadorian traditional lifestyle: *G. aequatoriale* is used as cuy (*Cavia porcellus*) fodder, *G. chilloense* is an antihemorrhagic, and *G. reptans* is used in wound healing [25].

**Figure 1:**
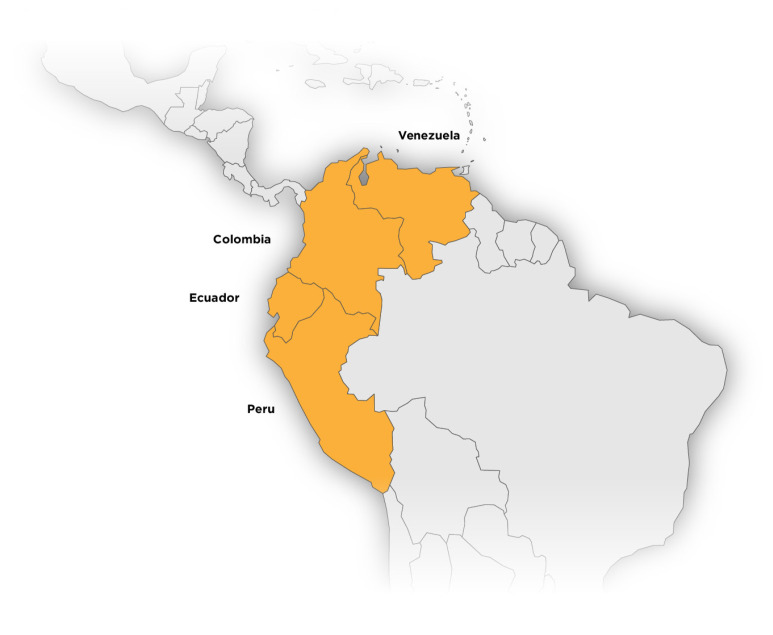
Distribution of *Geranium diffusum* Kunth.

**Figure 2:**
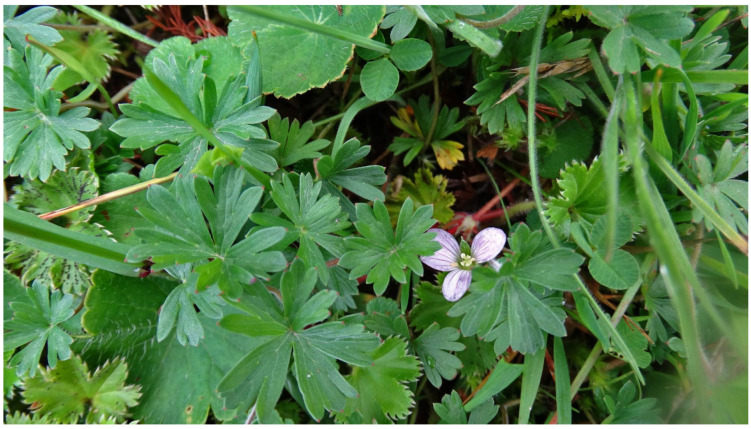
Flowering *Geranium diffusum* Kunth observed in Ecuador (CC-BY-4.0) [28].

*Geranium diffusum* Kunth. is a non-food medicinal herb, distributed in the Andes from western Venezuela to southern Peru, although it is most abundant in Ecuador and Peru [22]. The leaves of *G. diffusum* are densely covered with small, appressed hairs on both surfaces. It presents staminate fruits and clusters with a single flower; its petals are short and narrow, always glabrous, as are its filaments [22,26]. It is also known by its synonym *G. chimborazense* R. Knuth, under which it is listed as a vulnerable species [27]. The distribution of *G. diffusum* is shown in Figure 1, and the flowering plant is shown in Figure 2.

Some Spanish and Quichua vernacular names for *G. diffusum* are “Cancer”, “Agujilla”, “Ajotillo”, and “Chili chili” which are also given to other species in the genus (*G. ruizii*, *G. sessiliflorum*) and may thus lead to confusion among species. The English vernacular name “long-stalked cranesbill” is also confusing because it is primarily used for *G. columbinum*. The reported uses of *G. diffusum* are environmental, ornamental, medicinal, and dye [29,30]. It is noteworthy that one of the vernacular names of the species is “cáncer” although cancer does not figure—and is thus not treated—in the Saraguro ethnomedical system [7,31].

*G. diffusum* is a poorly studied medicinal plant. A bibliographic free-text search in the Dimensions database [32] using “Geranium diffusum” as the search term returned only eleven results, two of which focus on medicinal properties of plants [7,16].

The reported ethnomedical uses are few and general and are shown in Table 1: the plant is deemed non-toxic [33].

Common medicinal uses for plants of the genus include analgesic, antihemorrhagic, anticancer, anti-inflammatory, and antimicrobial [35,36]. The reported uses for *G. diffusum* are consistent with these.

Cancer is the leading cause of death in high-income countries, overtaking cardiovascular disease [37]. In 2020, there were 19.3 million new cancer cases and 10 million deaths due to this disease [38]. In spite of this, during the last decade, a general decrease in death rates due to cancer was seen, excepting liver cancer and lung cancer in women, in which death rates have increased [39]. This general reduction can be attributed, at least in part, to the development and approval of new treatments [40]. An estimated 25% of the treatments developed in the last decade are derived from natural products [41]. Therefore, the search for active secondary metabolites is important in making progress against the disease. Breast and colon cancers are among the most common cancers in Ecuador: breast cancer is the most common cancer in women, while colorectal cancer has the highest 5-year prevalence for both sexes [42,43].

Due to the presence of the species in traditional healing and the research gap in medicinal plants of the biodiverse Ecuadorian southern highlands it is important to advance towards scientific validation of the ethnomedical claims through a study of the species.

The objective of this study is to provide a phytochemical study of the methanolic extract of the species, with emphasis on flavonoid isolation and identification and to evaluate the activity of its extracts against selected cancer cell lines to validate ethnomedical uses and help reduce the research gap in non-food Ecuadorian traditional medicinal plants.

## 2. Results and Discussion

A total of 2049 g of *G. diffusum* aerial parts yielded 580 g dried plant material (28.4% yield), from which 37 g methanolic extract was obtained. This is a yield of 6.37%.

### 2.1. Phytochemical Composition

**Figure 3:**
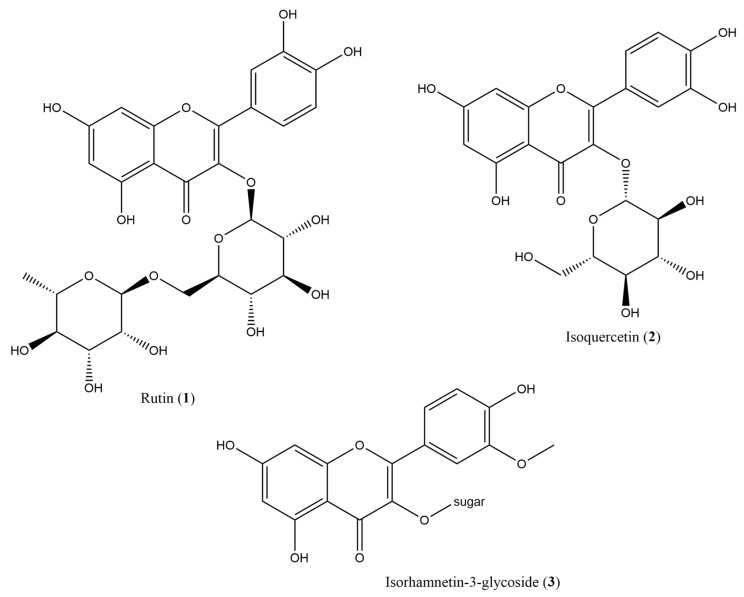
Isolated flavonoids from *G. diffusum* ethyl acetate fraction of the methanolic extract: rutin (**1**), quercetin-3-*O*-glucoside (isoquercetin) (**2**), and isorhamnetin-glycoside (**3**).

The phytochemical screening of the extract qualitatively revealed the presence of the following compound families in decreasing order. Very abundant: flavonoids. Abundant: proteins, terpenoids, and phenolics. Present: tannins and reducing sugars. Absent: fats, alkaloids, saponins, and quinones. Due to their abundance and bioactivity, flavonoid compounds were isolated and identified from the ethyl acetate fraction (Gd.P.EtOAc) of the extract using chromatographic methods. The isolated compounds are shown in Figure 3. The qualitative abundance nomenclature (+, ++, +++ for very abundant, abundant, and present) is in accordance with common practice in the field, for example, [44,45].

In addition, four compounds were presumably isolated but not identified due to the very small separation yields. The identified compounds are as follows:

Rutin (quercetin 3-*O*-rutinoside) (**1**). The compound was obtained as a yellow solid, with a RF of 0.5 in reverse phase TLC with MeOH:H_2_O 70:30 as eluent. The NMR signals were compared to those in the literature for confirmation and assignment [46,47]. The signals are the following:

^1^H–NMR (500 MHz, CD_3_OD, δ ppm, *J* in Hertz): 1.12 (d, *J* = 6.2 Hz, 3H, H6″′), 3.25–3.27 (m, 1H, H4″), 3.27–3.29 (m, 1H, H4″′), 3.3 (m, 1H, H5″), 3.4 (m, 1H, H3″), 3.5 (m, 1H, H2″), 4.52 (d, 1.4, 1H, H1″′), 6.20 (d, *J* = 2.1 Hz, 1H. H6), 6.39 (d, *J* = 2.1 Hz, 1H, C8), 3.64 (dd, *J* = 3.5, 1.4 Hz, 1H, H2″′), 7.67 (d, *J* = 2.1 Hz, 1H, H2′), 3.40 (t, *J* = 5.0, 3.6 Hz, 1H, H6″), 3.44 (dd, *J* = 6.2, 2.7 Hz, 1H, C5″′), 5.11 (d, *J* = 7.6 Hz, 1H, H1″), 3.81 (d, *J* = 9.8 Hz, 1H), 6.87 (d, *J* = 8.5 Hz, 1H), 3.54 (dd, *J* = 9.5, 3.5 Hz, 1H), 7.63 (dd, *J* = 8.5, 2.1 Hz, 1H).

^13^C NMR (125 MHz, CD_3_OD, δ ppm, (carbon number): 61.3 (5″), 77.3 (3″), 82.0 (2″), 86.6 (4″), 93.4 (8), 98.6 (6), 104.3 (10), 108.2 (1″), 115.1 (2′), 115.6 (5′), 121.6 (1′), 121.7 (6′), 133.5 (3), 145.0 (3′), 148.9 (4′), 157.6 (9), 159.1 (2), 162.6 (5), 165.4 (7), 177.3 (4).

Typical signals in **1** are the methyl group of rhamnose (H6″′) at 1.12 ppm, the anomeric proton of rutinose at 4.42 ppm (H″′), both in ^1^H, and the carbonyl in the aglycone moiety at 177.3 ppm in ^13^C, plus the usual flavonoid proton and aromatic carbon signals. The spectra and other NMR experiments for **1** can be found in Supplementary Material Figures S1–S3.

Isoquercetin (**2**). The compound was obtained as a yellow solid soluble in methanol, with a R_F_ of 0.55 in reverse phase TLC with MeOH: H_2_O 70:30 as eluent. The ^1^H NMR signals correspond to a quercetin glycoside (Figure S4 in Supplementary Material). The structure was validated by comparison with the literature [48].

^1^H–NMR (500 MHz, CD_3_OD, δ ppm, *J* in Hertz): 5.16 (d, *J* = 8, H1″), 6.20 (d, *J* = 2.10, 1H, H6), 6.40 (d, *J* = 2.10, 1H, H8), 7.84 (d, *J* = 2.19, 1H, C2), 6.86 (d, *J* = 8.49, 1H, C5′), 7.58 (dd *J* = 2.19, 8.49, 1H, H6′).

The anomeric proton signal is covered by the water signal. A 1D TOCSY experiment (Figure S5) shows the characteristic doublet with *J* = 8 Hz typical of the anomeric proton in pyranosyl rings.

Isorhamnetin-3-glucoside (**3**). The compound was obtained as a yellow solid, with a RF of 0.45 in reverse phase TLC with MeOH:H_2_O 60:40 as eluent. ^1^H NMR signals (Figure S6 in Supplementary Material) correspond to an isorhamnetin glycoside [49].

^1^H–NMR (500 MHz, CD_3_OD, δ ppm, *J* in Hertz): 3.64 (s, 3H, methoxyl in C3′), 6.21 (d, *J* = 2.0, 1H, H6), 6.38 (d, *J* = 2.1, 1H, H8), 7.34 (d, *J* = 2.1, 1H, H2′), 6.91 (d, *J* = 8.3, 1H, H5′), 7.31 (dd, *J* = 8.3, 2.1, 1H, H6′).

Flavonoid aromatic proton signals are consistent with isorhamnetin, particularly the methoxyl signal at 3.69 ppm, and there are sugar proton signals between 3 and 4 ppm. The recovered amount was insufficient to perform more NMR experiments and confirm the sugar structure.

Rutin is a well-studied flavonoid with one of the highest antioxidant activities in natural products, as well as antibacterial, antiprotozoal, antitumor, anti-inflammatory, antiallergic, antiviral, cytoprotective, vasoactive, hypolipemiant, antiplatelet, antispasmodic, and antihypertensive activity [46]. Rutin possesses proven in vitro and in vivo anticancer activity hampered by its reduced water solubility [50].

Two of the isolated compounds from *G. diffusum*, **1** and **2** are quercetin glycosides. Quercetin is among the most studied flavonoids because of its biological activity. Quercetin exhibits several structural characteristics consistent with its biological activity as an antioxidant, antibacterial, anticancer, and enzyme inhibition agent: 2,3 double bond, 4C carbonyl, and four hydroxyl groups in carbons 5,7,3′ and 4′ [51]. Compound **2** has proven anticancer activity, including breast, colon, and skin cancers [52,53].

Compound **3** is a glycoside of isorhamnetin, a monomethoxyflavonol. The methoxylation in C3′ is the only structural difference between quercetin and isorhamnetin. Isorhamnetin possesses ample biological activity, including vascular protection, neuroprotective, antithrombotic, hypoglycemic, antitumor, anti-inflammatory, lung protection, anti-osteoporotic, antioxidant, hepatoprotective, immunoregulator, antibacterial, and antiviral [54]. Isorhamnetin shows antitumor activity in a variety of cancers, including breast and colon cancer, through the inhibition of proliferation and induction of apoptosis [54,55]. Isorhamnetin is hydroxylated in C5, C7, and C4′, all of which enhance antibacterial activity, although 3′ methoxylation is considered to reduce the antibacterial activity in flavonoids [51]. The antidiabetic effect of flavonoids through inhibition of Dipeptidyl Peptidase-4 (DPP-4) is augmented by the 2,3 double bond, the C4 carbonyl group, and the 4′ hydroxyl, and decreased by a C3 hydroxylation and the methylation of the hydroxy group in C3′ [56]. Methylation of OH groups in flavonoids is correlated with an increase in anticancer activity, as is the number of hydroxyls [57]. The sole presence of antioxidant species in an extract does not imply beneficial biological activity, as, for example, extracts with high antioxidant activity can interfere with the activity of chemotherapeutic agents [58].

The isolated compounds are aligned with the flavonoid compound of other species of the *Geranium* genus. Quercetin derivatives are found in other species of the genus, and flavonoid content has been found to be highest in the methanolic extract in other species of the genus, for example, *G. robertianum* [35,59].

### 2.2. Citotoxicy on Colon Cancer Cells

The viability of three cell lines exposed to the methanolic extract and five solvent fractions of *G. diffusum* are shown below: RKO, SW613-B3, and HCT-116.

In the RKO and HCT-116 cell lines, after being exposed to the six fractioned extracts of *G. diffusum*, no viabilities of less than 50% were observed compared to the negative control.

**Figure 4:**
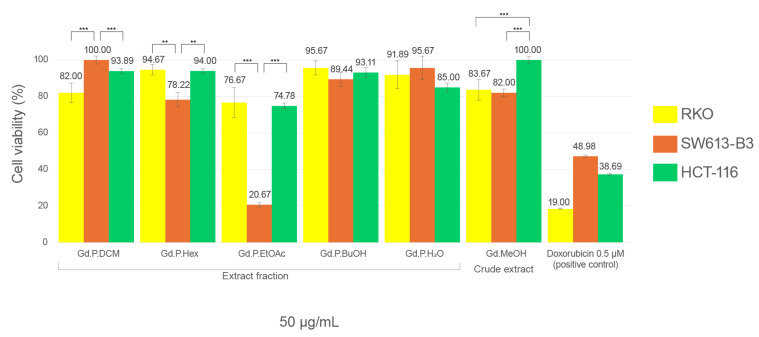
Viability of each of the cell lines exposed to each fraction of the methanolic *G. diffusum* plant extract for 48 h at 50 µg/mL, expressed as percentages of the average of three independent experiments with their respective standard deviation. Analysis using Anova post Tukey test: ** *p* < 0.05, *** *p* < 0.05.

**Figure 5:**
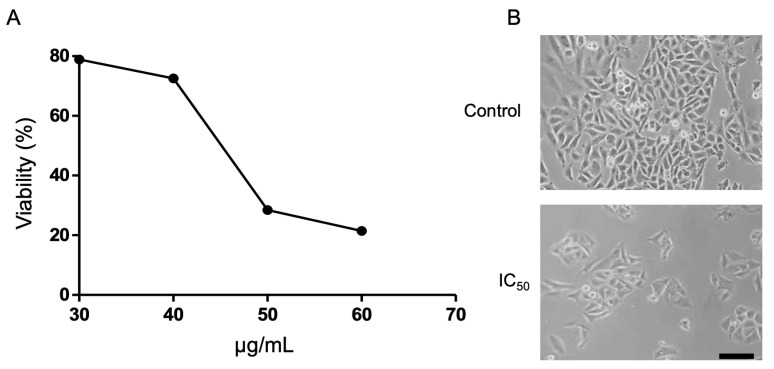
IC_50_ of more potent fraction: ethyl acetate. (**A**) Viability of the SW613-B3 cell line exposed for 48 h to increasing concentrations of the ethyl acetate fraction of the methanolic *G. diffusum* extract. (**B**) Cellular morphology after treatment with the IC_50_ of the extract. A decrease in the cell population is observed. Scale bar 50 μm.

On the other hand, in the SW613-B3 cell line, the ethyl acetate fraction presented a significant cytotoxic effect with an IC_50_ = 44.47 ± 1.02 μg/mL. In the other fractions and the methanol extract, viability of over 70% is evident. The dichloromethane (DCM) fraction exerts a greater effect on RKO and HCT-116 cell lines than on SW613-B3 cells. The hexane fraction presented lower viability in the SW613-B3 cell line, while the methanolic fraction showed greater viability in the HCT-116 cells, showing significant differences in the viabilities of these extracts among cell lines (Figure 4 and Figure 5).

Several extracts of species in the genus *Geranium* have been reported for cytotoxic activity on tumor lines [60,61,62], with effects similar to those observed in this study. The effect observed with the most active fraction could be related to the presence of rutin, quercetin, and isorhamnetin, which in other studies have shown cytotoxic activity on colon and breast cancer tumor lines [54,63,64,65,66].

### 2.3. Genotoxicity

**Figure 6:**
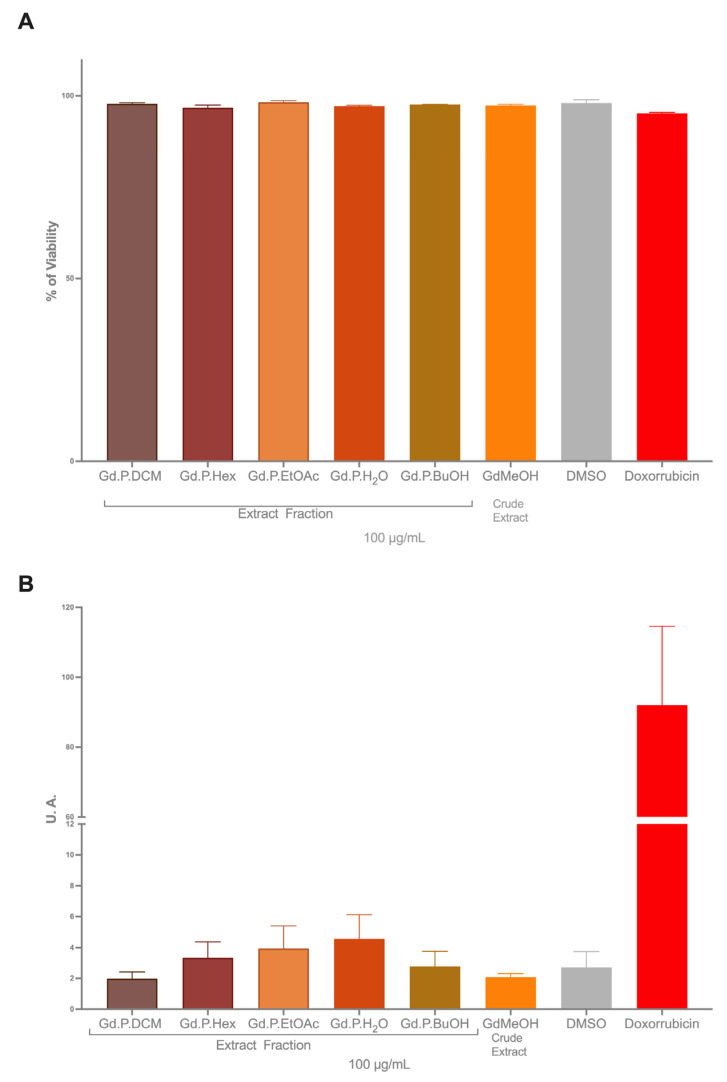
Genotoxicity assessment of CHO-K1 cells exposed to 100 µg/mL of methanolic *G. diffusum* extracts and fractions for 24 h. (**A**) Cell viability. (**B**) DNA damage assessed by comet assay (tail moment). DMSO (0.25%) and Doxorubicin (2 µM) were used as negative and positive controls, respectively. Data represent the mean ± SD of three independent experiments.

An ideal antitumor substance should be selective, inducing death in tumor cells without affecting normal cells at the same dose. Additionally, regarding genotoxicity, it is desirable for a substance, whether natural or synthetic, to maintain cell viability above 70% at the tested doses. In our study using CHO-K1 cells as a model for normal cells, we found that *Geranium diffusum* extracts and fractions provide higher cell viability compared to tumor cells (Figure 6A). These results allowed us the use of higher doses (100 µg/mL) to assess the genotoxicity of the extract, confirming its safety. Tail moment analysis, a parameter that correlates with the length and intensity of the comet tail, indicated that neither the methanolic extract nor the fractions induced a statistically significant increase in DNA damage when compared to the control group (Figure 6B).

The comet assay in the same tumor cells could provide information about the mechanism of cell death; however, our study is focused on the safety of the extract consumption [67,68]. That is why the cell model was changed using a recognized model for general genotoxicity assays such as CHO-K1 cells. The interesting thing about our study was finding a different dose between normal and tumor cells. We have found that it does not generate an increase in the comet tail at a dose of 100 µg/mL (twice the dose tested in tumor cells) like other extracts of the genus [61,62]. Also, due to the presence of quercetin in the extract, a compound that is found in other cell models has shown a basal increase in damage in the comet assay [69,70,71]; however, when mixing this flavonoid with compounds that damage DNA, it is capable of reducing the damage and presents protective properties [72,73,74]. That is why it is interesting to continue studying the extracts of *G. diffusum* and its chemopreventive activities.

## 3. Materials and Methods

### 3.1. Chemicals, Reagents, and Cell Lines

Hexane, dichloromethane, ethyl acetate, silica gel 60 (0.063–0.0200 mm), LiChroprep RP-18 (40–63 µm), TLC silica gel 60 F_254_, and TLC silica gel 60 RP-18 F_254_s aluminum sheets were purchased from Merck, Darmstadt, Germany. Sephadex LH-20, *n*-butanol, methanol, glutamic acid, albumin, powdered milk, Dragendorff reagent, caffeine, vanillin, sucrose, glucose, RPMI medium, fetal bovine serum (FBS), DMSO, and doxorubicin were purchased from Sigma-Aldrich, St. Louis, MO, USA. All solvents and reagents were of analytical grade.

Ethidium bromide and MTS colorimetric assay were purchased from Promega (Madison, WI, USA). EDTA, NaCl, and Tris base were purchased from Invitrogen (Carlsbad, CA, USA). NaOH was purchased from Fisher Scientific (Pittsburgh, PA, USA). L-Glutamine, Antibiotic-Antimycotic (Penicillin G, Streptomycin, and Amphotericin B), trypsin, and HAM F-12 medium were purchased from GIBCO (Grand Island, NY, USA).

The colon carcinoma cell lines RKO (CRL-2577), HCT-116 (CCL-247), and the *Cricetulus griseus* cell line CHO-K1 (CCL-61) were purchased from ATCC, and SW613-B3 was provided by Dr. Ivanna Scovassi, CNR–IGM, Pavia, Italy.

### 3.2. Plant Collection and Extracts

**Figure 7:**
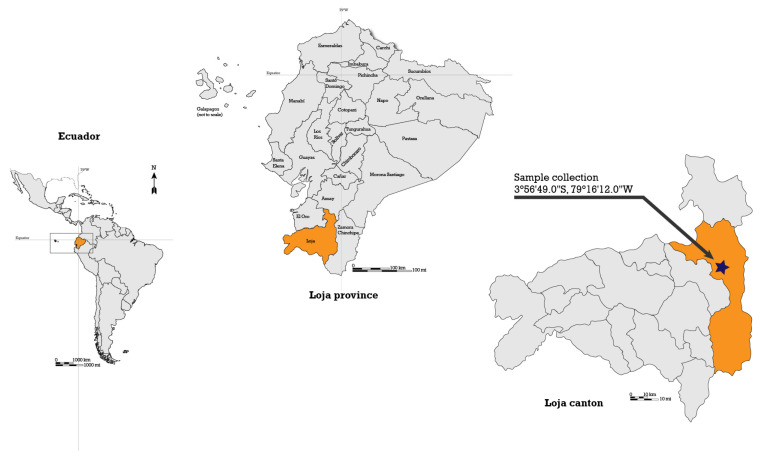
*G. diffusum* collection area.

A total of 2049 g of *G. diffusum* aerial parts were collected on 15 November 2017, from a field near Loja-Chuquiribamba Road at coordinates 03°56′49″ S, 79°16′12″ W at 2500 m above sea level (Figure 7). The specimen was collected in compliance with the Framework Contract MAE-DNB-CM-2016-0048 dated 20 September 2016, between UTPL and the Ecuadorian Ministry of the environment, water, and ecological transition (MAE). Once the species was identified by Dr. Fani Tinitana and a sample was deposited in the UTPL Herbarium (voucher HUTPL9363), the rest of the collected material was dried under airflow at 37 °C for seven days until constant weight was attained.

The dry plant material was coarsely ground, and the extract was obtained through static maceration for 3 days with 5 L methanol, shaking the container twice a day, and concentrated to dryness on a rotary evaporator (Buchi R210, Flawil, Switzerland). The extraction procedure was repeated three times on the plant samples to yield the *G. diffusum* methanolic extract which was weighed, labeled, and stored at −18 °C prior to use. The methanolic extract was chosen over the aqueous extract for its usually greater biological activity [75,76].

### 3.3. Phytochemical Screening

Phytochemical screening of the crude methanolic extract was performed according to standard procedures following the methods of Mandal et al. and Miranda-Martínez [77,78]. Protein detection was performed via the Biuret copper-complex formation test, with egg albumin, powdered milk, and glutamic acid used as positive controls [79]. Reducing sugars were detected via the Fehling test, with sucrose and glucose as positive controls [80]. Lipid detection was performed through the Sudan fat-soluble dye, with vegetable oil as positive control [81]. Alkaloids were tested for through the Dragendorff potassium tetraiodobismuthate test, using caffeine as positive control [82]. Terpenoids were detected by the Lieberman Burchard acetic anhydride test, using Argentatin B as positive control [83]. Flavonoids were screened by the Shinoda magnesium and hydrochloric acid test: the positive control was hesperidin [84]. Saponins were detected by the foam test, using grated raw potato as positive control [85]. Quinones were detected using the Bornträger test, with hydroquinone as positive control [86]. Phenolic compounds were detected with the FeCl_3_ assay. Vanillin was used as the positive control [87].

### 3.4. Fractioning of the Extract

**Figure 8:**
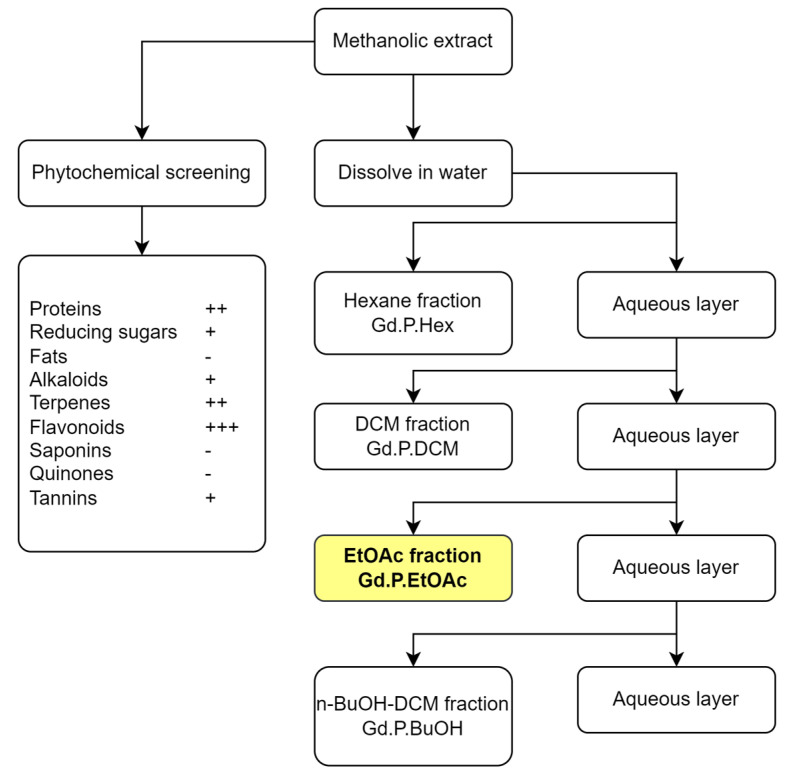
*G. diffusum* methanolic extract fractionation scheme. Phytochemical screening qualitative results are: - absent; + present; ++ somewhat abundant; +++ very abundant.

The methanolic extract of *G. diffusum* was divided into fractions that contain compounds of different polarities with the purpose of evaluating the biological activity of the fractions, using liquid–liquid extraction. Then, 20 g of the extract was dissolved in 400 mL distilled water in a 2500 mL separatory funnel and extracted sequentially with equal volume of solvents of increasing polarity, all of them immiscible with water, hexane, dichloromethane, ethyl acetate, and *n*-butanol/dichloromethane, with the purpose obtaining fractions enriched in compounds of increasing polarity, following the procedure of Silva-Rivas et al. [88]. The fractioned extracts were concentrated on a rotary evaporator (Buchi R210, Flawil, Switzerland) and stored at −18 °C pending use. The fractioning scheme is presented in Figure 8.

From the methanolic extract, five fractions were obtained by liquid–liquid separation (Table 2). The ethyl acetate fraction was subject to further chromatographic separations because of its highest activity against SW613-B3 cells, with emphasis in flavonoid identification, because they are the most studied compounds in the phenolic compound class, which is in turn the most studied class among phytochemicals, due to their wide biological activity, and because they are known to be soluble in the ethyl acetate fraction of methanolic extracts [76,89].

### 3.5. Secondary Metabolite Isolation and Identification

The separation of the ethyl acetate fraction consisted of progressive chromatographic separations with different stationary phases and isocratic and solvent gradients using the method described by Silva-Rivas et al. with slight modifications [88]. Fractions are named F1, F2, and so forth, and fractions of these are F1F1, F1F2, etc.

An amount of 4 g of the ethyl acetate fraction were separated to isolate and identify compounds. A 370 mm length by 25 mm diameter open column was packed with direct phase silica gel 60 as the stationary phase, with a 1:5 fraction extract-to-silica ratio. A gradient elution was performed with solvent mixtures of increasing polarity: CH_2_Cl_2_:MeOH (90:10), CHCl_3_:MeOH:H_2_O (75:23:02), CHCl_3_:MeOH:H_2_O (71:25:04), and CHCl_3_:MeOH:H_2_O (65:25:04). A total of 49 fractions were obtained, evaluated by thin-layer chromatography and united by chromatographic similarity, resulting in eight fractions (F1 to F8). Fractions F2, F5, and F6 were selected for further purification based on their chromatographic separation potential [90].

Fraction F2 was separated using Flash chromatography (Buchi Reveleris^®^ PREP, Flawil, Switzerland) with direct phase silica 60 on an 80 mm length × 25 mm diameter column in a sample to silica proportion of 1:200. The elution was performed with a solvent mixture of CH_2_Cl_2_:MeOH from 95:5 to 50:50. Then, 144 separations were collected and regrouped based on TLC similarity into five fractions F2F1 to F2F5. Fraction F2F4 was further purified using the same Flash chromatography procedure with isocratic Hex:EtOAc 20:80 as eluent. Next, 103 separations were obtained and combined into 10 fractions based on TLC similarity. Fraction F2F4F1 yielded what appeared to be three compounds, but due to the low yield, they were not identified.

Fraction F6 was eluted using the same Flash chromatography setup as F2 in a EtOAc:MeOH solvent gradient from 100:0 to 0:100. One hundred separations were obtained and combined into seven fractions based on TLC similarity. Fraction F6F3 was separated using reverse phase TLC sheets in MeOH:H_2_O 80:20. The largest fraction (F6F3F1) was recovered through filtration but 1.3 mg of the sample size was insufficient for ^1^H NMR identification. Fraction F6F1 was eluted in a 200 mm length by 15 mm diameter Sephadex LH-20 microcolumn using MeOH as eluent. From the 90 separations obtained, four fractions were obtained after combining based on TLC similarity. Further purification was performed on fraction F6F1 using preparative direct phase TLC with an EtOAc:MeOH:H_2_O 90:7:3 eluent. Two compounds were obtained; however, due to the low yield, the ^1^H NMR spectrum was inconclusive. We were unable to identify the compounds.

**Figure 9:**
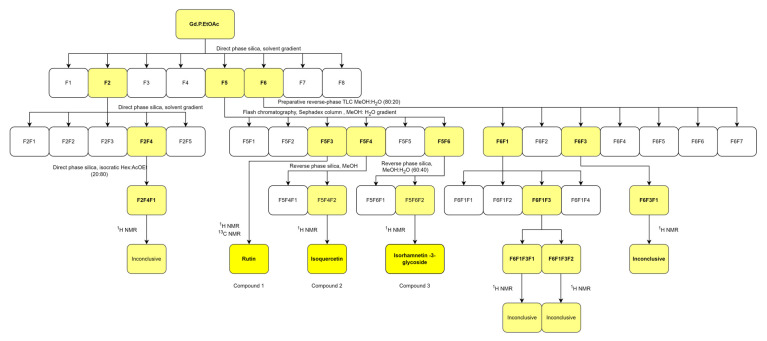
Isolation and identification scheme from the ethyl acetate fraction (Gd.P.EtOAc) of the methanolic extract.

Fraction F5 was separated through flash chromatography (Buchi Reveleris^®^ PREP, Flawil, Switzerland) on a standard 24 cm length by 4 cm diameter column using Sephadex LH-20 with a MeOH–MeOH:H_2_O (80:20) gradient at 8 mL/min with a total elution time of 245 min. The 72 resulting separations were combined based on TLC similarity into six fractions (F5F1 to F5F6). Fraction F5F3 yielded rutin, identified by ^1^H (500 MHz) and ^13^C (125 MHz) on a 500 MHz Bruker NMR (Billerica, MA, USA). Fraction F5F4 was eluted on LiChroprep RP-18 on a standard 8 cm length by 1.5 cm diameter column using MeOH as eluent and yielded two fractions. Fraction F5F4F2 corresponds to isoquercetin, determined by ^1^H NMR spectroscopy. Fraction F5F6 was separated through preparative reverse-phase TLC using MeOH:H_2_O (80:20) as eluent. Fraction F2F6F2 was identified as isorhamnetin-3-glycoside by ^1^H NMR. The isolation scheme is summarized in Figure 9.

To summarize, compound (**1**) was isolated as F5F3, (**2**) was isolated as F5F4F2, and (**3**) was isolated as F5F6F2. This implies that the flavonoid-rich fraction is eluted with medium to high polarity solvent mixture from the original gradient, and again, the more polar elution on Sephadex.

### 3.6. Cell Viability

#### 3.6.1. Cell Culture

The colon carcinoma cell lines RKO, HCT-116, and SW613-B3, were used as a biological model for cytotoxicity. The cells were cultured and maintained in RPMI medium supplemented with 10% fetal bovine serum (FBS), 2 mM L-Glutamine, Antibiotic-Antimycotic (Penicillin G 100 U/mL, Streptomycin 100 μg/mL, and Amphotericin B 0.25 μg/mL), at 37 °C in a humid atmosphere with 5% CO_2_ [68].

#### 3.6.2. Cell Viability Through MTS Testing

To determine the cytotoxic effect of the extracts on human tumor cell lines, the MTS colorimetric assay was applied, which measures cell survival and proliferation through the metabolic reaction of tetrazolium salts or MTS to formazan by the mitochondrial enzymes NADH or NADPH from living cells [91].

For each cell line, 3200 cells/100 µL/well were seeded in a 96-well plate. After 24 h of incubation, the cells were exposed to 50 µg/mL of methanol extract and the five solvent fractions. In addition, the blank, negative control was considered using the solvents and positive control was considered using Doxorubicin at a concentration of 0.5 µM during an incubation period of 48 h. Four hours before finishing the treatment, 20 µL of the MTS reagent was applied. The absorbances were monitored in a BioTek spectrophotometer (EPOCH2), at a wavelength of 490 nm. The data obtained were processed and transformed into percentages considering the negative control as 100% cell viability.

#### 3.6.3. Inhibitory Concentration

To establish the inhibitory concentration 50 (IC_50_), increasing concentrations were used according to the fraction of ethyl acetate that had a cytotoxic effect in the MTS assay; less than 50% viability. As mentioned previously, 3200 cells/100 µL/well were seeded in a 96-well plate, after 24 h of incubation the SW613-B3 cell lines were exposed to the Gd.P.EtOAc fraction at increasing concentrations of 30, 40, 50, 60 µg/mL for 48 h. Four hours before the end of the treatment, the MTS reagent (20 µL) was added and the same procedure as previously explained was carried out.

#### 3.6.4. Morphological Analysis

To determine the effect induced by the fraction (Gd.P.EtOAc) that showed the highest level of cytotoxicity in the SW613-B3 cell line, we proceeded to seed 40,000 cells in a 12-well plate and after 24 h each line was exposed to the IC_50_ of the extract and all its fractions, in addition to positive (Doxorubicin 2 µM) and negative controls (DMSO). Then, 48 h after being treated, the cells were observed and photographed in the Axioskop 2 plus optical microscope (Zeiss, Göttingen, Germany).

### 3.7. Genotoxicity

#### 3.7.1. Cell Culture

The *Cricetulus griseus* cell line CHO-K1 was employed as a biological model. Cells were cultured and maintained in HAM F-12 medium, supplemented and maintained like other cells.

#### 3.7.2. Treatment and Viability FDA-Ethidium Bromide

A total of 8000 cells/100 µL/well were seeded on a 96-well plate and incubated for 18 h. The treatment with the extract and its fractions was applied at a concentration of 100 µg/mL. Additionally, DMSO was used as a negative control at a concentration of 0.1%, and doxorubicin was used as a positive control at a concentration of 0.5 µM.

Cells were trypsinized with 0.25% trypsin and collected with their culture medium. The supernatant was discarded, and the cell pellet was resuspended in 500 µL of supplemented HAM F-12 medium and centrifuged again under the previously described conditions. An aliquot of the cell suspension was used to assess cell viability, while the remaining cell suspension was used for the comet assay. The entire procedure was performed at 4 °C. Cell viability was determined using the methodology described by Bailon-Moscoso et al. (2016). A solution was prepared with fluorescein diacetate (FDA) at a concentration of 5 mg/mL and ethidium bromide at a concentration of 0.2 mg/mL. A total of 200 cells, both live and dead, were counted under a fluorescence microscope Axioskop 2 plus (ZEISS, Germany) using filter N°4 and a 40× objective [92].

#### 3.7.3. Comet Assay

The comet assay was performed as described by Bailon-Moscoso et al. [68], with minor modifications, which are described below. Cells were embedded in agarose slides for lysis solution preparation containing 1% Triton X-100, 2.5 M NaCl, 100 mM EDTA, and 10 mM Tris base at pH 10. Slides were incubated in lysis solution for 24 h at 4 °C in the dark. Subsequently, electrophoresis was performed at 25 V and 300 mA for 20 min in a buffer containing 300 mM NaOH, 1 mM EDTA, and pH > 13 for 20 min. Slides were neutralized with 0.4 M Tris at pH 7.5 and then fixed with methanol for subsequent comet analysis. Slides were stained with 60 µL of ethidium bromide (1.5 µg/mL) and analyzed using a ZEISS-Axioskop 2 plus fluorescence microscope with a 40× objective. One hundred cells per slide were counted. Finally, the data were analyzed using Comet Assay IV software (Perceptive Instruments Ltd., Bury Saint Edmunds, UK) [93].

### 3.8. Statistical Analysis

All assays were performed in triplicate in three independent experiments. Statistical analysis was performed on GraphPad Prism version 10.1.1 (San Diego, CA, USA). The viability results are shown from mean values with their respective standard deviations through ANOVA statistical analysis, using the Dunnet test. A three-parameter regression was used to calculate IC_50_. Genotoxicity was assessed using the Kruskal–Wallis post hoc test. Differences were considered statistically significant at *p* < 0.05.

## 4. Conclusions

The phytochemical analysis of the methanolic extract of *Geranium diffusum* suggests the presence of terpenes, flavonoids, and proteins in the plant extract, with flavonoids as the primary active constituents, with rutin, isoquercetin, and isorhamnetin-3-glycoside as the major compounds. The ethyl acetate fraction demonstrated pronounced cytotoxicity against SW613-B3 cells, suggesting its therapeutic potential and consistent with the activity of the aglycones of the identified compounds. No genotoxicity was found in CHO-K1 cells, which could suggest a chemoprotective effect that warrants further study. These findings are a reminder of the importance of traditional medicinal plants as reservoirs of untapped bioactive molecules with potential applications in cancer treatment. Further studies are required to explore pharmacokinetics, activity in other cell lines, in vivo efficacy, and possible synergies of these flavonoids and other compounds.

The present study, as a first, is limited in its scope, emphasizing the methanolic extract, flavonoid isolation, and activity against RKO, SW613-B3, and HCT-116 cell lines. Ensuing studies should address these limitations, by performing a wider set of extracts that enable the identification and isolation of a variety of compound classes, and to generalize the findings of this study on a variety of cell lines.

This work contributes to the scientific understanding of traditional medicinal plants, especially the poorly studied ones, and also makes a case for the conservation and sustainable use of native Ecuadorian medicinal biodiversity and knowledge.

## Figures and Tables

**Table 1:** Ethnomedical uses of *Geranium diffusum* Kunth.

Use	Plant Organ	Country	Preparation/ Administration	Ref.
Stomach pains	NS	Ecuador	Leaf infusion	[34]
Postpartum infections, gangrene	Whole plant	Ecuador	Crushed plant/Oral	[16]
Wound-healing, analgesic	NS	Peru	NS	[29]

NS: not specified.

**Table 2:** Weight and yield of the *Geranium diffusum* methanolic extract fractions.

Fraction	Weight (g)	Yield (%)
Hexane	7.45	29.8
Dichloromethane	0.078	0.31
Ethyl acetate	4.18	16.7
Butanol	4.45	17.8
Aqueous	2.35	9.4

## Data Availability

All data are presented in the document.

## Supplementary Materials

The following supporting information can be downloaded at: https://www.mdpi.com/article/10.3390/plants14050777/s1. Figure S1: ^1^H NMR spectrum of compound (**1**)—Rutin; Figure S2: ^13^C NMR spectrum of compound (**1**)—Rutin; Figure S3. COSY spectrum of Rutin (**1**). Figure S4. ^1^H NMR spectrum of isoquercetin (**2**). Figure S5. TOCSY spectrum of isoquercetin (**2**) showing the anomeric carbon proton with its distinctive coupling constant (*J* = 8 Hz). Figure S6. ^1^H NMR spectrum of isorhamnetin-3-glucoside (**3**).
