# Supplementary material for: Phytochemical Study, Cytotoxicity, and Genotoxicity of the Methanolic Extract of Geranium diffusum Kunth
Source: Plants (Basel). 2025 Mar 3;14(5):777. doi: 10.3390/plants14050777 (PMC11902229; doi:10.3390/plants14050777)
Supplement: Supplementary file 1 [file plants-14-00777-s001.zip › plants-3408078-supplementary.pdf]

Article

Phytochemical study and cytotoxicity of the methanolic extract of *Geranium diffusum* Kunth — Supplementary material

Juan Carlos Romero-Benavides, Tatiana Añazco-Loayza, Anabel Correa-Sinche, Andrea Alvarez, Luis Miguel Guamán-Ortiz, Rodrigo Duarte-Casar, and Natalia Bailon-Moscoso

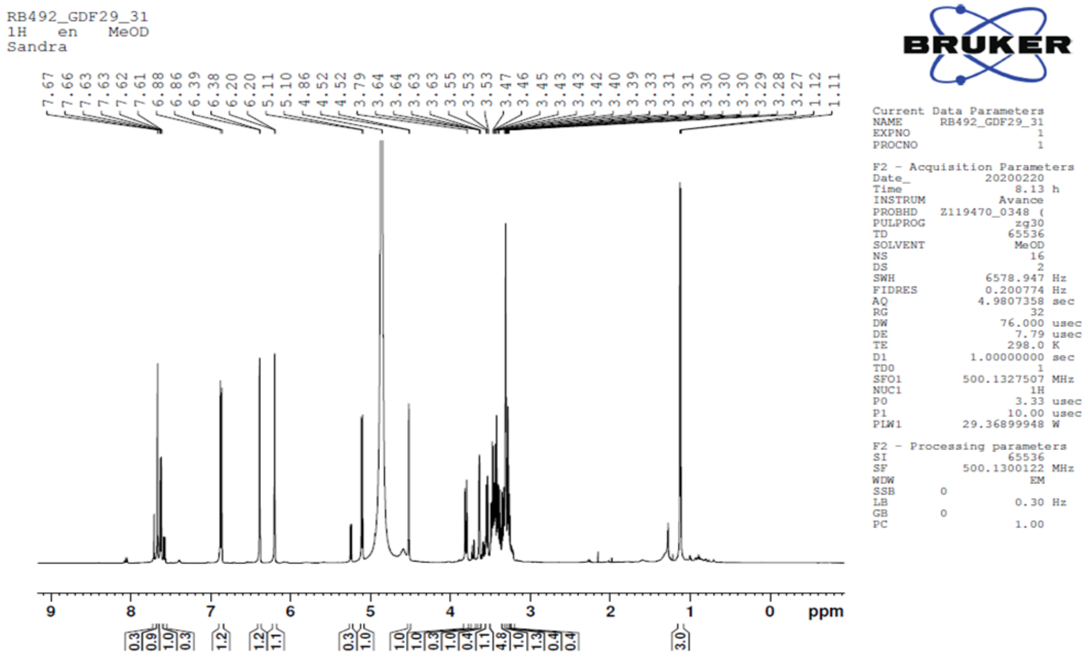

Figure S1: ^{1}H NMR spectrum of compound (**1**) - Rutin

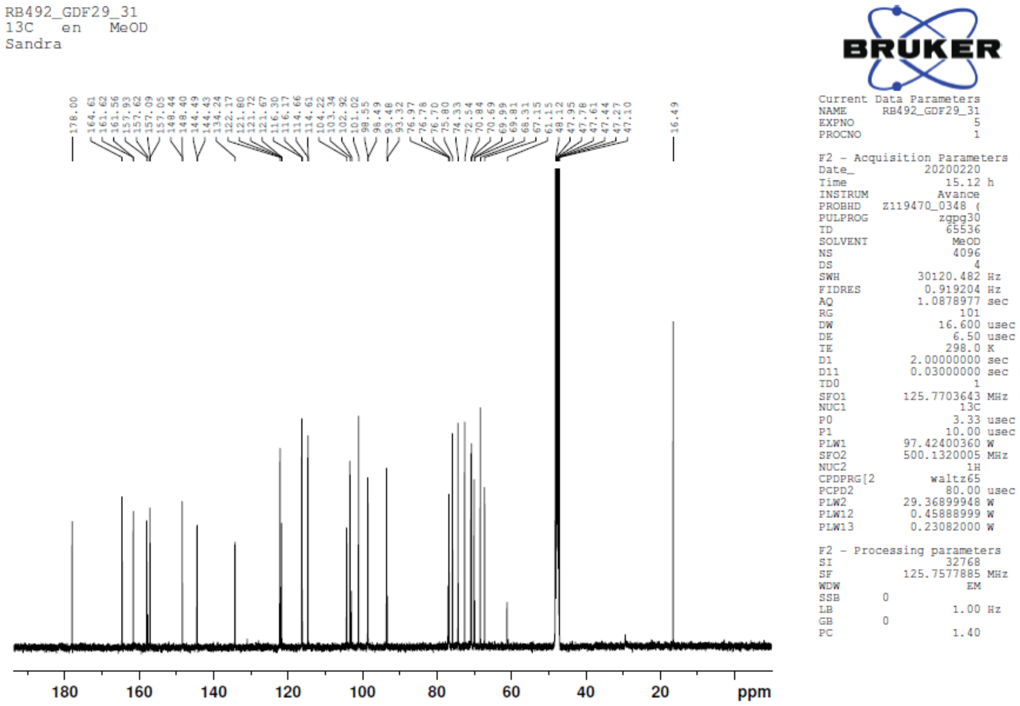

Figure S2: ^{13}C NMR spectrum of compound (**1**) - Rutin

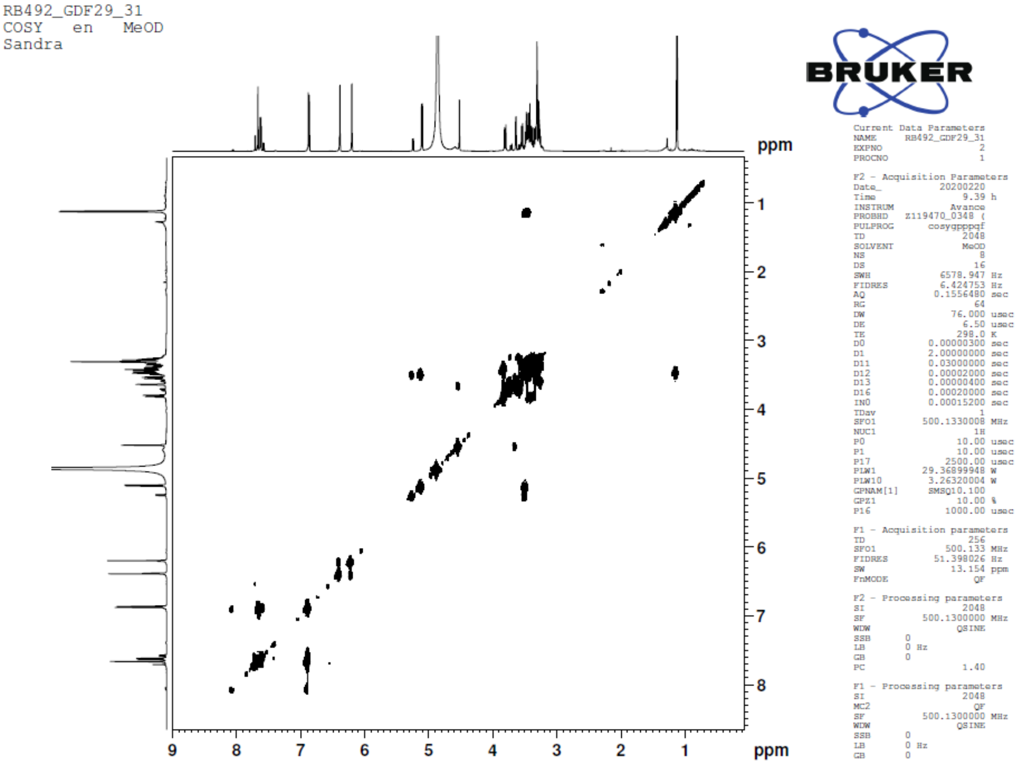

Figure S3. COSY spectrum of Rutin (**1**).

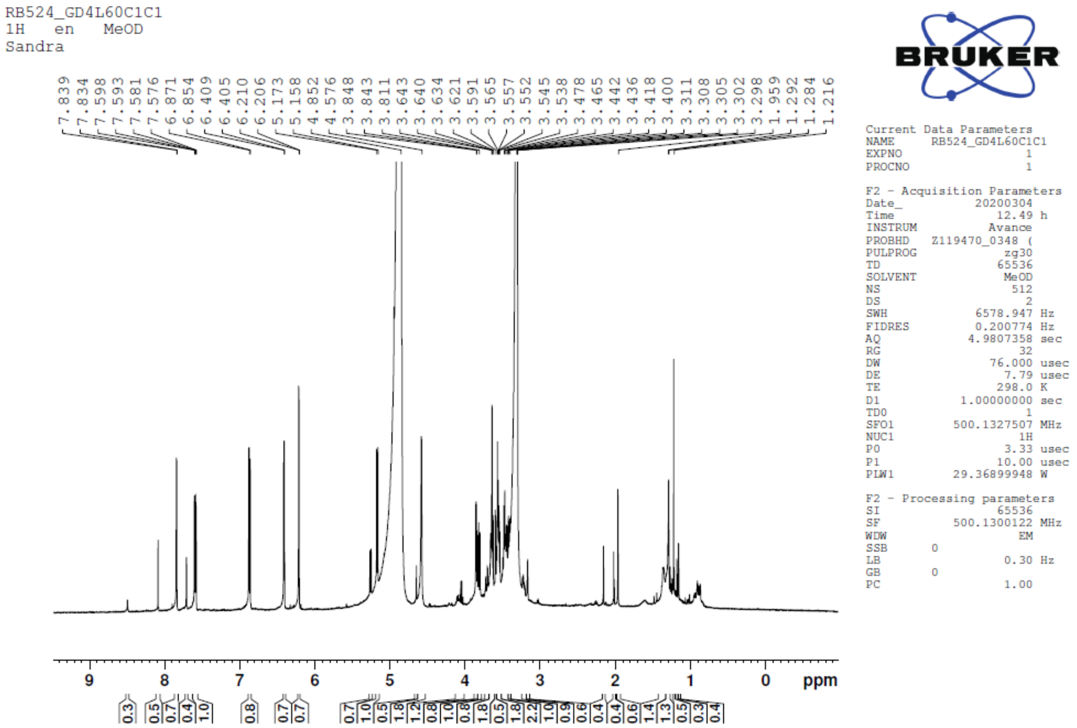

Figure S4. ^{1}H NMR spectrum of isoquercetin (**2**).

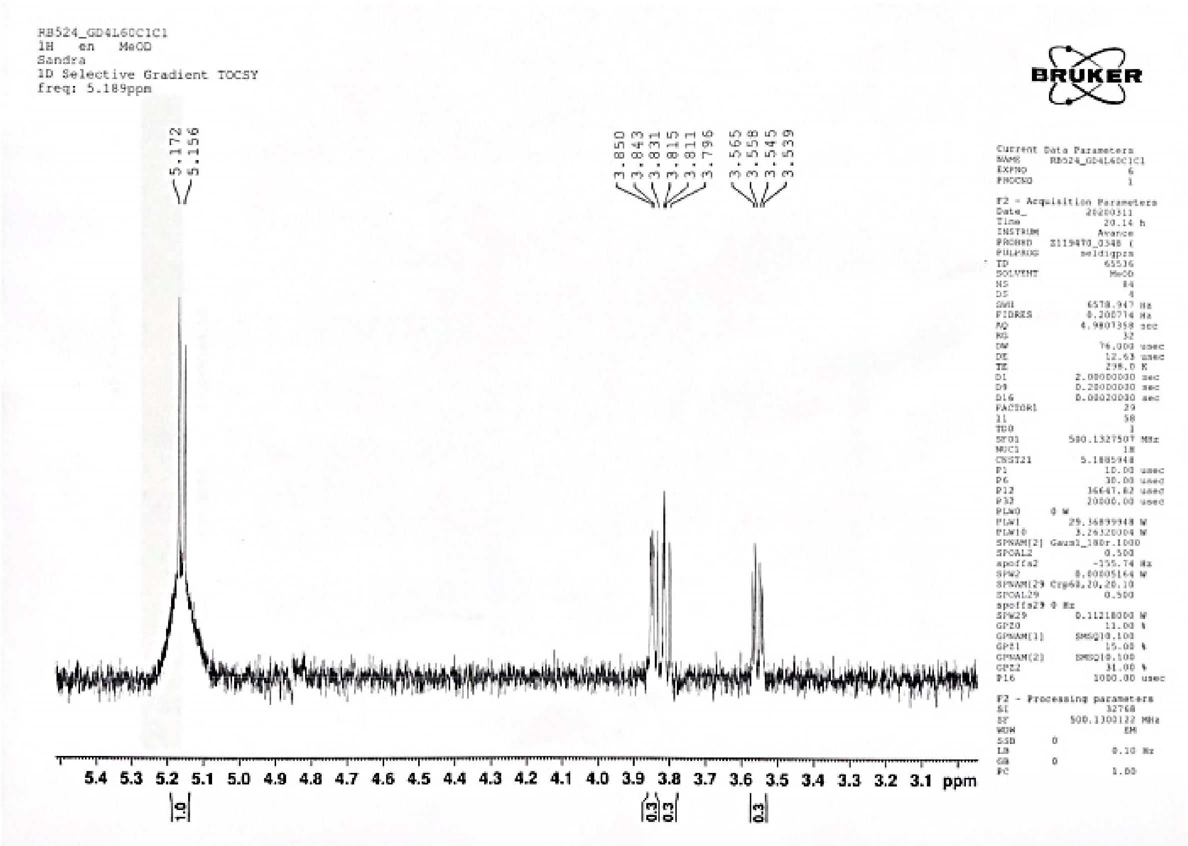

Figure S5. TOCSY spectrum of isoquercetin (**2**) showing the anomeric carbon proton with its distinctive coupling constant (*J* = 8Hz).

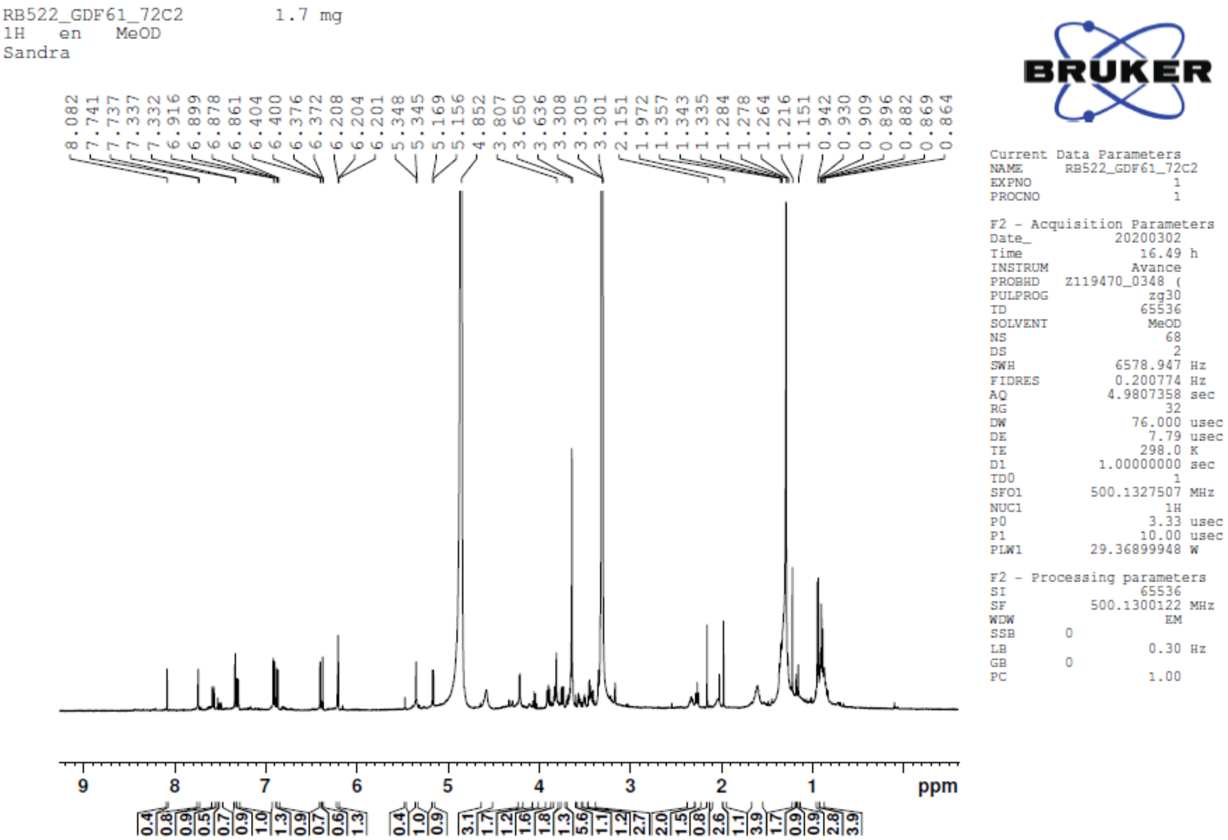

Figure S6. ^{1}H NMR spectrum of isorhamnetin-3-glucoside (**3**).
